# A Comparison of Volatile Components Across Native Australian Mentha (Lamiaceae)

**DOI:** 10.3390/plants15050778

**Published:** 2026-03-03

**Authors:** Trevor C. Wilson, Paul I. Forster, Daniel J. Duval, Joseph J. Brophy

**Affiliations:** 1 National Herbarium of NSW, Botanic Gardens of Sydney, Locked Bag 6002, Mount Annan, NSW 2567, Australia; trevor.c.wilson@botanicgardens.nsw.gov.au; 2 Queensland Herbarium & Biodiversity Science, Department of the Environment, Tourism, Science & Innovation, Brisbane Botanic Gardens Mt Coot-tha, Toowong, QLD 4066, Australia; paul.forster@detsi.qld.gov.au; 3 SA Seed Conservation Centre, Botanic Gardens & State Herbarium of Adelaide, North Terrace, Adelaide, SA 5000, Australia; daniel.duval@sa.gov.au; 4 School of Chemistry, University of New South Wales, Sydney, NSW 2052, Australia

**Keywords:** *Mentha*, GC/MS, volatile components

## Abstract

*Mentha* are historically important regarding their volatile oils. Since limited accounts exist for Australian species, we document the variation in volatiles across all Australian *Mentha* species, using the GC/MS of pentane extractions from leaf samples of replicate populations for all known species. Oil yields were consistently poor (<0.2% *w*/*w*) for freshly dried and herbarium specimens. Many species uniformly had high percentages of volatiles characteristically attributed to *Mentha* (*viz.* Menthone, Pulegone); yet, others—consistently or variably—lacked them. *Mentha australis* had the highest concentrations of menthone (25%), isomenthone, (9%) and pulegone (24%), and *M. diemenica* had menthone (32.5%) and pulegone (29.8%). Extracts from *M. grandiflora* from herbarium specimens produced weak traces, high in menthone and pulegone. *Mentha satureioides* had the highest menthone (20–30%) and pulegone (22–28%) in populations across the extent of its range; yet, an entirely different chemotype was identified from eastern New South Wales that contained limonene (17%), 1,8-cineole (19%), and α-terpineol (8%). *Mentha laxiflora* consistently exhibited limonene (27%); yet, the levels of the other main components (e.g., menthone, β-pinene, germacrene-D, and bicyclogermacrene) varied across populations. *Mentha atrolilacina* exhibited the most unique oil profile, with main components consisting of linalool (21%), β-caryophyllene (14%), germacrene-D (14%), and bicyclogermacrene (23.7%). Commercial samples of *M. satureioides* were found only to be the chemotype high in limonene (17%) and 1,8-cineole, which warrants further taxonomic research and caution for the industry seeking mint flavours from Australian sources.

## 1. Introduction

**Figure 1:**
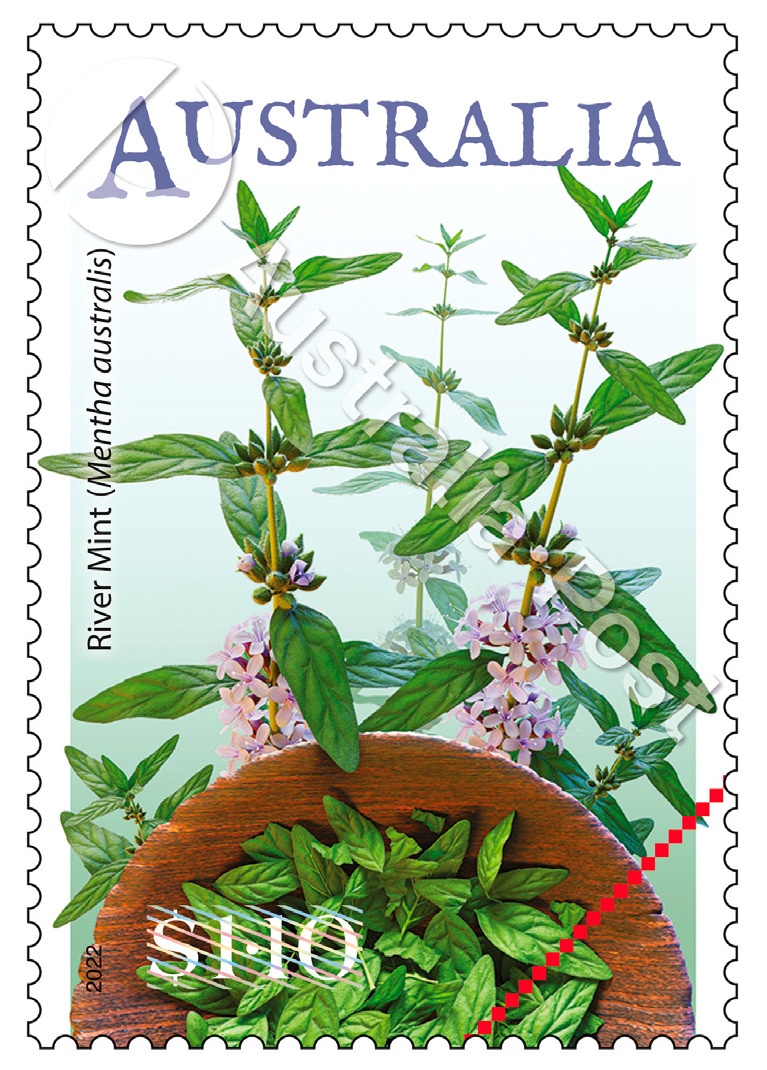
An example of the celebrated use of *Mentha* in Australia, as acknowledged through the “Bush Seasonings” stamps series issued by Australia Post in 2022 (© Australia Post 2022, Illustration Gavin Ryan).

Lamiaceae, or the ‘mint family’, are a well-recognised group useful for culinary, medicinal, and ornamental uses. Likely the most celebrated of its members is *Mentha* L., commonly known as ‘Mint’, which are fragrant rhizatomous herbs of generally damp, open habitats [1]. The aromatics in *Mentha* are most celebrated for flavouring food and drinks, but extracts from these plants have a broad range of applications including as antimicrobials, biocides, and medicines (Figure 1) [2,3,4,5,6,7,8,9]. Commercial oil production is largely restricted to only a few species or cultivated hybrids, including *M. citrata* Ehrh. (*M. aquatica* L. var. *citrata* (Ehrh.) Fresen), *M. canadensis* L., *Mentha* × *piperita* L. (Peppermint), *M. pulegium* L. (Pennyroyal), *M. spicata* L. (Native Spearmint), and *M.* × *gracilis* Sole (Scotch Spearmint). The popular flavours these plants produce (e.g., mint, pennyroyal, peppermint, and spearmint) is due to their high concentrations of menthols and pulegone, menthofurans, and carveols [10].

**Figure 2:**
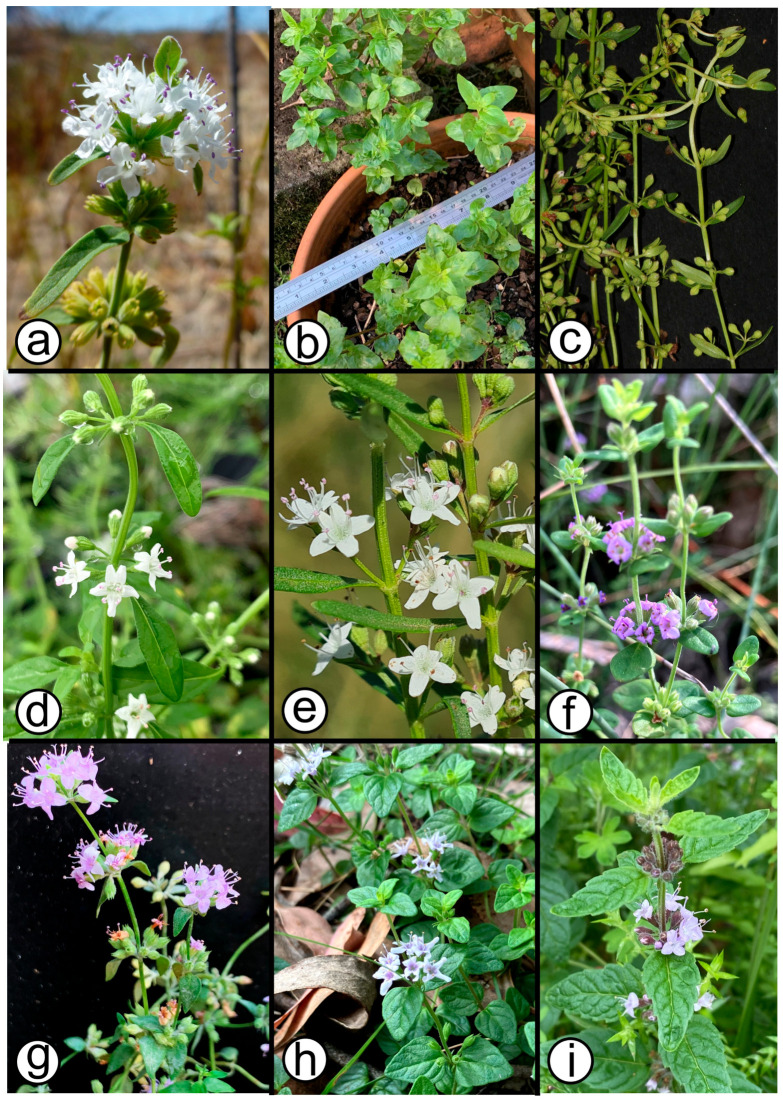
Diversity of *Mentha* species across Australia: (**a**) *M. australis*, from Murtho Native Forest Reserve, SA; (**b**) *M. australis* of unknown provenance, cultivated from IndigiGrow Native Plants Nursery, Matraville, NSW; (**c**) *M. satureioides* cultivated from collection DJD3644 made at Kanmantoo, SA; (**d**) *M. satureioides* associated with T.C.Wilson 1019, from Wategora Nature Reserve, Sydney, NSW; (**e**) *M. satureioides* associated with P.I.Forster PIF48899, from Jimbour West, Darling Downs, Qld; (**f**) *M. atrolilacina* cultivated from collection DJD1017 made at Honans Native Forest Reserve, SA; (**g**) *M. diemenica* associated with DJD1768, cultivated from Honans Native Forest Reserve, SA; (**h**) *M*. sp. aff. *laxiflora* associated with Duval s.n., cultivated from Track to Dry Creek campground, north of Donovans, SA; and (**i**) *M. laxiflora* from Tallaganda National Park, NSW. Photos: D.Duval (**a**,**f**,**h**); J.Brophy (**b**); T.C.Wilson (**c**,**d**,**g**); G.Leiper (**e**); and OneTapir from iNaturalist (**i**).

**Figure 3:**
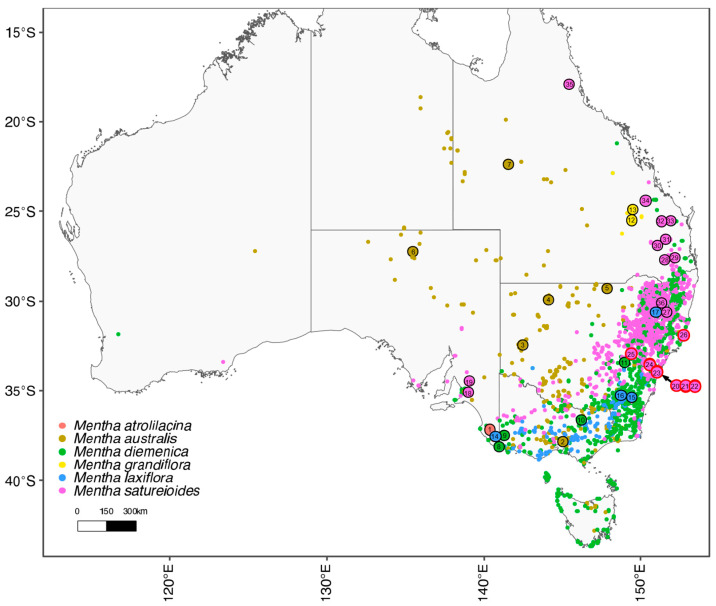
Distribution of native *Mentha* species across Australia. Distributions are generated from Atlas of Living Australia data (http://www.ala.org.au, accessed on 13 June 2025), with larger outlined circles identifying the location of specimens sampled in this study, and the numbers referring to the source as identified in Table 1. Black versus red outlines for *M. satureioides* are indicative of chemotypes 1 and 2 reported from the results of this study, respectively.

The taxonomy of *Mentha* has had a legacy of instability [11], with 25 species and 15 hybrids currently recognised [12]. Widespread polyploidy associated with cryptic species appears to have complicated an accurate delineation of the component taxa. It is being revealed through genomic tools how a mismatch between the genotype and morphotype has undermined traditional species identification and complicated the extent of our knowledge about its biodiversity [13,14]. Within Australia, there are six naturally occurring, endemic *Mentha* species (Figure 2): *M. atrolilacina* B.J.Conn & D.J.Duval, *M. australis* R.Br., *M. diemenica* Spreng., *M. grandiflora* Benth., *M. laxiflora* Benth., and *M. satureioides* R.Br [15,16,17]. These species form a distribution that spans the eastern half of the continent (Figure 3). *Mentha pulegium* is a seventh species naturalised throughout southeastern Australia. Although this exotic species is morphologically similar to *M. diemenica* and *M. satureioides* (i.e., ‘*M. satureioides* group’ *sensu* Conn [15]), it can readily be distinguished by the densely flowered verticillasters (vs. sparsely flowered verticillasters) and hairy inner corolla tube (vs. glabrous inner corollas tube) [15,18]. According to the most recent phylogenetic study of *Mentha* infrageneric relationships, Australian species (i.e., *M. diemenica* and *M. satureioides*) and the New Zealand *M. cunninghamii* Benth. form a clade that is sister to Eurasian and American species [19]. One study has so far provided empirical morphological evidence about the distinctiveness between *M. australis* and *M. diemenica* [20], but no other test of species boundaries or phylogenetic study for Australasian species has been made.

The most comprehensive treatment for Australian *Mentha* is shared in the Flora of New South Wales and Flora of Victoria [15,16]. Both of these flora account for four of the six Australian species (i.e., *M. australis*, *M. diemenica*, *M. laxiflora,* and *M. satureioides*). Relative to the ‘*M. satureioides* group’, *M. australis* and *M. laxiflora* are readily identified by having more or less spreading hairs on the inner surface of their calyx (vs. villous hairs) and have few- to many-flowered inflorescences (i.e., up to seven per axil vs. three per axil). *Mentha grandiflora* is similar to *M. australis*, but has broader leaves and larger flowers [21,22], and *M. atrolilacina* is similar to the ‘*M. satureioides* group’ [18] but has cordate leaves (vs. ovate or elliptic) and flowers with inserted anthers (vs. exserted anthers).

A persisting challenge in the taxonomy of Australian *Mentha* continues to be the ‘*M. satureioides* group’ (*sensu* Conn [15,16]), as it is unclear whether it represents distinct species or extremes of a morphological continuum. Declaring this ambiguity, for the interim, Conn [15,16] distinguished *M. satureioides* from *M. diemenica* as having nearly glabrous branches (vs. hairy branches), glabrous outer calyx (vs. hairy calyx), narrow-elliptic to linear-elliptic leaves (vs. ovate, often angular-ovate and rarely narrow-ovate or elliptic leaves) with an entire margin (vs. sometimes having a margin with distant shallow teeth, if not entire). Conflict in the recognition of species for this group is apparent between state floras. For example, the National Herbarium of NSW (NSW) identifies specimens collected from Queensland as either *M. satureioides* and *M. diemenica*; yet, the Queensland Herbarium (BRI) recognises only *M. satureioides* as indigenous to the Queensland state.

Unsurprisingly, use of Australian *Mentha* has been similar to that in the northern hemisphere. Decoctions of *M. australis* were considered by indigenous Australians as beneficial for coughs and colds as well as for an abortifacient [23]. After the arrival of Europeans to Australia, accounts of *M. australis* (or ‘River Mint’) identified the scent of its oil as similar to peppermint, but coarser [2]—it has since been used for flavouring (e.g., desserts and drinks), cold and cough medicine, insect repellent, and antioxidant or anti-inflammatory compounds for skincare [3]. The characterization of phenolics (higher-molecular-weight components, not volatile ones) [24] and antioxidants [25] have been intensively investigated for this species; yet, the oil chemistry has been quantified by only one study using ex situ material in Saudi Arabia [26]. The essential oil of *M. laxiflora* was recorded as similar to that of peppermint, and that of *M. grandiflora* is strongly fiery, bitter, and unpleasantly nauseous to taste [2]. *Mentha satureioides* (syn. *Mentha gracilis*) was originally described as similar to peppermint, and reported to have a slight admixture of pennyroyal and be quite disagreeable and acrid [2]—its uses have included being a diuretic, diaphoretic, insect repellent, or for regulating menstrual cycles [2]. For this species, the most intensive essential oil study of any Australian *Mentha* distilled 100 lbs. of ‘wilted’ *M. satureioides* from Dalby Qld, obtaining 93 g of oil (0.2%) that was described to resemble the “pennyroyal oils of commerce” [27]. Later, the essential oil composition of *M. satureioides* (originally identified as *M. diemenica*—see taxonomy above) from Didcot, Qld (approximately 100 km north of the Dalby location) was reconfirmed as having significant amounts of menthone and pulegone [28].

The examination of replicate material in the quantification of volatiles is a sensible idea, granted that individuals even within a population of the same species can have a different oil composition. Early investigations of eucalyptus essential oils suggested that the composition of the oil was typically constant within the same species [29]—the typical leaf oil make up for *Eucalyptus* [30], as well as *Melaleuca* [31], can be defined as including 1,8-cineole (~60–70%), α-pinene (~10%), limonene (~8%), and α-terpineol (~5%). However, it has been discovered that trees of the same species, even growing beside one another, can produce different compounds (in several families including Myrtaceae), and thereby are described as separate chemotypes (e.g., [31,32,33]). Nonetheless, distinct oils and oil profiles continue to assist with the identification and description of new taxa (*Eucalyptus* and others) [27,34,35,36,37], and the miniaturization of methods (working with mgs of leaf material) to quantify and qualify them is enabling other uses of herbarium samples to assist with species description [38,39].

In this study, we aim to provide the first comprehensive study of oil quantification for all Australian *Mentha*—we reanalyse previously sampled species, as well as provide the first account of oil composition for *M. atrolilicana* and *M. laxiflora.* Using GC/MS (gas chromatography/mass spectrometry) Total Ion Current (TIC) traces, we sampled the fresh material of most species, comparing relative rather than absolute quantities of compounds. Furthermore, sampling was augmented using herbarium material to include several populations across a species’ distribution in recognition of the unresolved taxonomy of *Mentha* [15,16], and the likelihood that several chemotypes may exist within a species. Additionally, through sample provision from multiple Australian horticulture suppliers, the wild type was also compared with industry stock. We ask the following questions: what are the chemotypes for each species, do they vary within species, and do they compare between samples sourced from commercial suppliers and with a known provenance?

## 2. Results

### 2.1. Mentha atrolilacina

**Figure 4:**
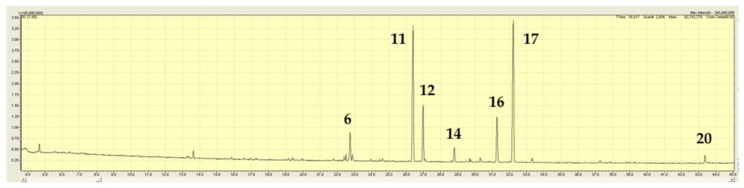
TIC trace on a polar column for *Mentha atrolilacina* (sample JB6181, Table 1). A number above a peak refers to a specific volatile compound listed in Table 2.

**Figure 5:**
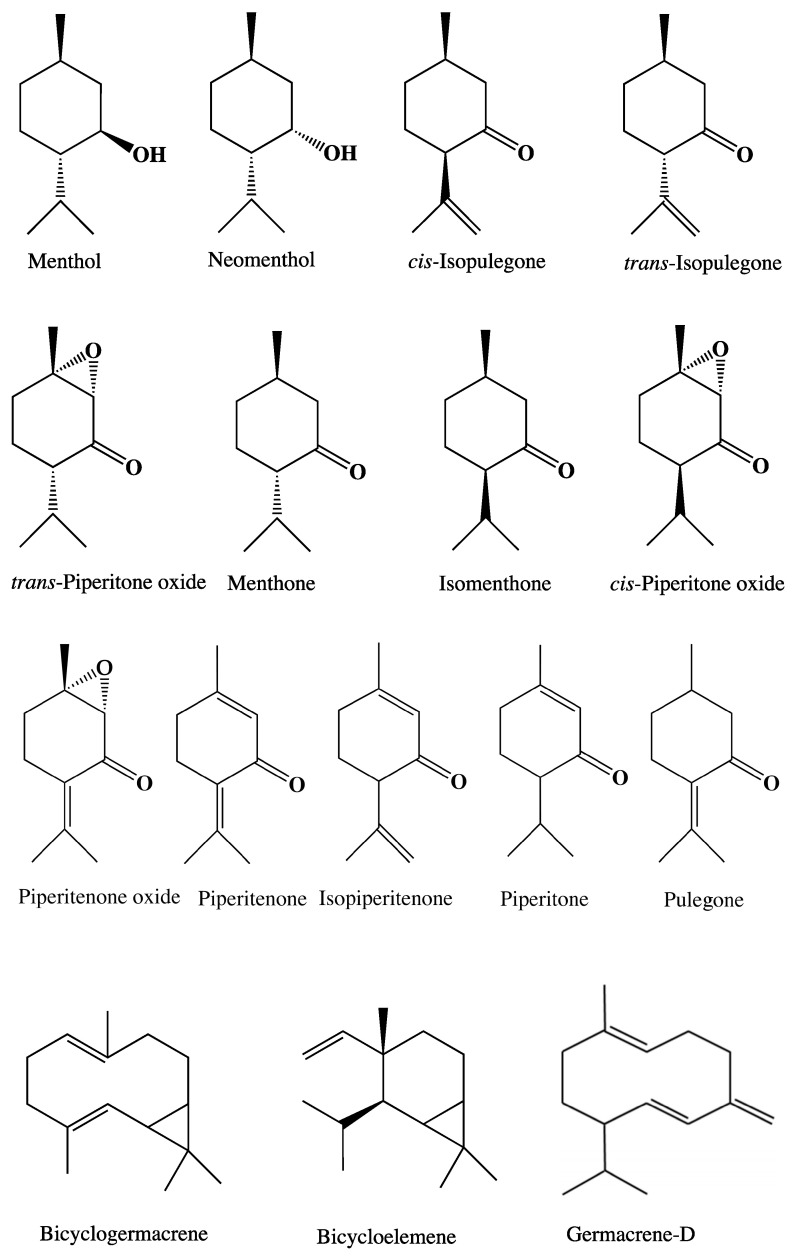
Structures of some compounds, mainly monoterpenes, that are described in this study.

*Mentha atrolilacina* GC/MS TIC traces demonstrated a mixture of volatiles containing four major components, the majority being sesquiterpene hydrocarbons. These were linalool (21.2%), β-caryophyllene (14.1%), germacrene-D (13.3%), and bicyclogermacrene (23.7%), (Figure 4; Table 2). Also present in amounts between 1–5% were γ-elemene, α-copaene, aromadendrene, δ-cadinene, and germacrene-D-4-ol (see Figure 5 for conformation of some of these compounds). There were more components present in amounts < 1%.

### 2.2. Mentha australis

**Figure 6:**
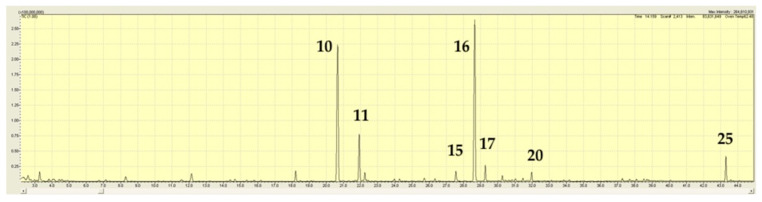
TIC trace on a polar column for *Mentha australis* extract from a commercial sample with unknown provenance (sample JB6174, Table 1). A number above a peak refers to a specific volatile compound listed in Table 3.

The major volatile components in the fresh *M. australis* extract were menthone (32%), isomenthone (11%), and pulegone (31%); see Table 3 and Figure 6, below. These components were accompanied by lesser amounts of α-pinene and β-pinene (1–1.5%), hex-2-enal (1.5%, isomer not determined), 3-hexene-1-ol (2.2%, isomer not determined), neomenthol (2.3%), menthol (3.1%), bicyclogermacrene (1.9%), and germacrene-D-4-ol. One individual plant from Melbourne, Victoria (JB6180) contained less menthone (20%) than pulegone (70%). From the extracts of herbarium samples, menthone, pulegone, and isomenthone were detected as prominent components in the extracts (Table 1).

### 2.3. Mentha diemenica

**Figure 7:**
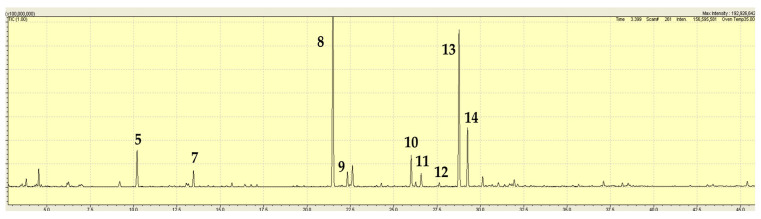
TIC trace on a polar column for *Mentha diemenica* of Honans Native Forest Reserve, South Australia (sample JB6209, Table 1). A number above a peak refers to a specific volatile compound listed in Table 4.

Fresh extracts of *M. diemenica* from Honans Native Forest Reserve, SA (JB6209) (see Table 4, Figure 7) contained menthone (32.5%) and pulegone (29.8%) in high amounts, and a somewhat high amount of menthol (9.7%) (Table 4, Figure 7). Herbarium extracts similarly had the highest amounts of menthone and pulegone, except one population from Victoria (JB6204) which was missing pulegone. Also matching the fresh extract were other compounds detected from some herbarium specimens including isomenthone, neomenthyl acetate, menthone, isomenthone, and menthol (Table 1).

### 2.4. Mentha grandiflora

Our extractions were solely based on herbarium specimens. Our results identified the major components of pulegone and menthone (Table 1).

### 2.5. Mentha laxiflora

**Figure 8:**
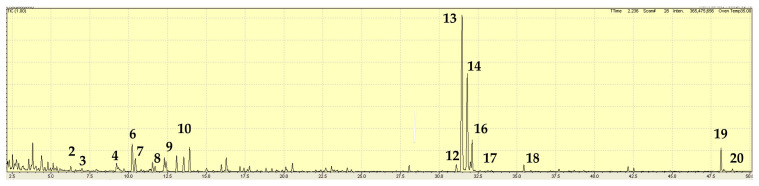
TIC trace on a polar column from the extract of *Mentha laxiflora* from Tallaganda National Park, New South Wales (sample JB6231). A number above a peak refers to a specific volatile compound listed in Table 5.

The volatiles, as major components in *M. laxiflora,* differed among the extracts of fresh specimens and herbarium specimens. The extraction of fresh material from Tallaganda National Park (JB6231) showed piperitone (24.9%) and *cis*-piperitone epoxide (30.4%) as major components. Other significant components were *trans*-piperitone epoxide (7.4%), limonene (9.0%), 1,8-cineole (3.5%), *p*-cymene (4.1%), thymol (5.2%), and bicyclogermacrene (2.1%) (Table 5, Figure 8).

**Figure 9:**
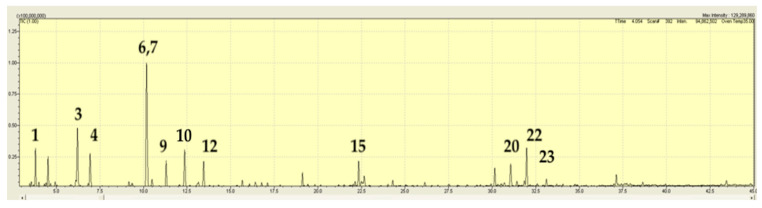
TIC trace on a polar column from the extract of *Mentha laxiflora* from road to Dry Creek Campground, north of Donovans, South Australia (sample JB6182). A number above a peak refers to a specific volatile compound listed in Table 6.

The extract from the South Australian *M.* sp. aff. *laxiflora* (JB6182) was dominated by limonene (27.4%), with significant amounts of the monoterpenes α-pinene (5.8%), β-pinene (11.3%), sabinene (6.3%), γ-terpinene (7.2%), and the olefinic 2-hexenal (4.4%, isomer not identified), and the principal sesquiterpenes being germacrene-D (4.5%) and bicyclogermacrene (7.1%) (Table 6, Figure 9).

Extracts from both herbarium specimens provided limonene; however, plants from the Goodradigbee River population also contained α-copaene (similar to Dry Creek) and β-caryophyllene, whereas, from the Warrabah National Park, the population showed 1,8-cineole, menthone, and isomenthone being present (Table 1).

### 2.6. Mentha satureioides

**Figure 10:**
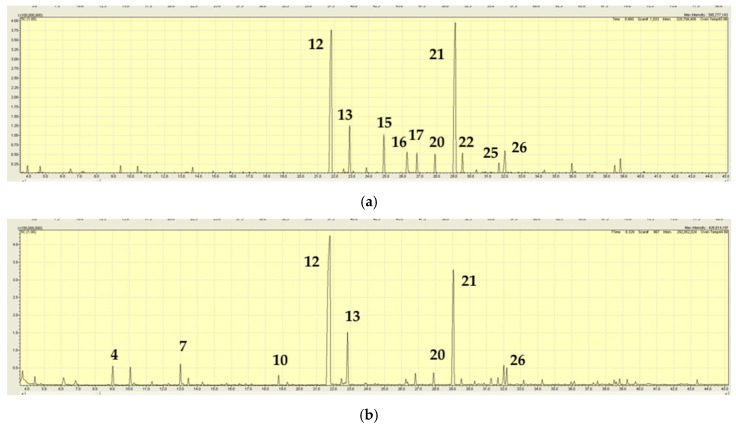
TIC traces on a polar column for *Mentha satureioides* from populations at opposite ends of its distribution: (**a**) Jimbour West, Queensland (JB6185); and (**b**) Kanmantoo, South Australia (JB6183). A number above a peak refers to a specific volatile compound listed in Table 7.

*Mentha satureioides* in Queensland and South Australia (Table 7, Figure 10) generally represent opposite ends of the species distribution. Comparing the fresh extracts from both ends of the distribution, the major components were menthone (22.5, 36%) and pulegone (22.5, 12.1%), respectively. These were accompanied by lesser amounts of isomenthone (8.7, 6.7%), neomenthyl acetate (7.2%), menthyl acetate (4.7%), *cis*-isopulegone (3.5%), *trans*-isopulegone (1.4%), piperitone (5.3, 2.8%), bicyclogermacrene (2.2%), and piperitenone (2.4%). Most other fresh and herbarium extractions were similar in their major volatiles, including menthone, and, to a lesser extent, pulegone—in herbarium specimens from northern New South Wales and northern Queensland, menthone was accompanied with either piperitone oxide and piperitenone oxide (former), or neomenthyl acetate and menthyl acetate (latter) instead.

**Figure 11:**
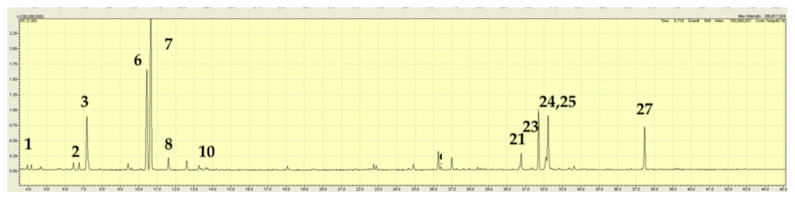
TIC trace on a polar column *Mentha satureioides* from Wategora Reserve, NSW (sample JB6216, Table 1). A number above a peak refers to a specific volatile compound listed in Table 8.

The fresh extracts sourced from Sydney, New South Wales (NSW) yielded a different set of volatiles, representing a second chemotype (Table 8, Figure 11). The extract was dominated by limonene (17.3%) and 1,8-cineole (19.2%), with sabinene (9.4%), α-terpineol (8.1%), and bicyclogermacrene (9.1%) being the other major components. Neomenthyl acetate and menthyl acetate (totaling 3.7%) were the only components in common with the fresh extracts of the first chemotype demonstrated above. Other fresh extracts from Sydney populations, Taree, all commercial supplies of unknown provenance, and herbarium specimens from Glenbrook and near Kerrs Creek similarly had major amounts of limonene and 1,8-cineole (Table 1).

## 3. Discussion

This is the first comprehensive account of the volatile compounds for Australian *Mentha* species, and the first account of the volatile compounds for *M. atrolilacina* and *M. laxiflora.* We found that the levels of menthone or pulegone (or close derivatives), which are characteristic economically useful volatiles attributed to the genus, were not consistently high across all species (e.g., *M. atrolilacina*), nor even within some species (e.g., *M. satureioides*). This alludes to the potential for chemotypes in Australian *Mentha* species, or, perhaps, to the idea that there are species that have not yet been identified.

### 3.1. Species with High Menthone and Pulegone Oil Profiles

We found that, across our replicated sampling of populations, *M. australis*, *M. diemenica*, and *M. grandiflora* are consistently high in levels of menthone (25–40%) and pulegone (25–40%) as dominant components. In addition to menthone and pulegone, there are lesser amounts of related compounds, both in the oxidation state and functional groups. For the profiles of *M. grandiflora* and *M. diemenica*, these are supplemented by *trans*-piperitone oxide and piperitenone oxide and other oxidised compounds based on piperitone (in all of these structures, there is oxidation at the carbon adjacent to the isopropyl group).

The uniform oil profile of *M. australis* with distinctively high amounts of menthone and pulegone documented in all wild populations sampled in this study corresponds to the early reports (i.e., the 1861 Dublin Exhibition) that mention the species has ‘pennyroyal’ smells [2]. Fresh extracted commercially supplied samples, which have the highest amounts of menthone followed by pulegone, match closely with all samples we analysed from herbarium specimens. Fresh wild-sourced material from the Melbourne population [JB6180] does have a disproportionately larger amount of pulegone than menthone. However, such a difference in the oil profile is comparably small in relation to the only other study of isolated extracts from ex situ *M. australis* samples [26], which found distinctively high amounts of linalool, and little to no trace of menthol. The fact that our relatively consistent results from a widespread Australian sampling also matches the earliest anecdotal evidence of *M. australis* volatiles suggests the anomalous account is due to a misidentification. However, this is difficult to test given that the previous study did not disclose the herbarium records or the provenance of its material.

**Figure 12:**
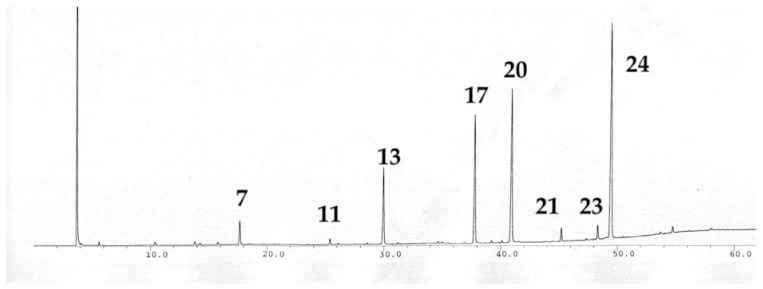
FID (Flame Ionisation Detector) trace on a polar column of the leaf essential oil of *Mentha grandiflora* from Precipice National Park, Queensland from the study by Brophy et al. [22]. A number above a peak refers to a specific volatile compound listed in Table 9.

The high levels of menthone and pulegone match previous studies made for *M. grandiflora* from Precipice National Park, Queensland (see Table 9, Figure 12). From our only sample of *M. grandiflora*—a herbarium specimen collected in Palmgrove National Park—we obtained two extremely small peaks, which were of comparable intensity to the impurities in our pentane solvent, matching the two distinct menthone and isomenthone peaks shown by [22]. The poor signal from our extraction likely did not exhibit other volatiles similar to those found in smaller amounts from the previous study (which were obtained by steam distillation on a much larger amount of fresh leaf) [22], such as the piperitenone oxide (36.2%), *trans*-piperitone oxide (21.4%), pulegone (19.1%), menthone (9.7%), piperitenone (1.7%), bicyclogermacrene (2.1%), and limonene (3.5%) (Table 9, Figure 12).

The high levels of menthone and pulegone we recorded from fresh *M. diemenica* (JB6209) match the results of previous extractions of cultivated material originally sourced from Canberra, ACT [28]. Similar to this study, we also note, in lower quantities (yet still above 1%), neomenthyl acetate, isomenthone, *cis*-isopulegone, and myrcene. However, unlike this study, we also detected limonene, *cis*-isopulegone, and myrcene. Despite being weak in signal, herbarium specimens similarly yielded the highest levels of menthone and pulegone.

### 3.2. Two Putative Chemotypes for Mentha satureioides

Most *M. satureioides* populations we examined have high levels of menthone and pulegone, in the same reasonably high quantities as *M. australis, M. grandifolia,* and *M. diemenica*. This includes all our sampling from Queensland and South Australia, and one from New South Wales (i.e., JB6207), whose quantities range around approximately 32% menthone and 25–44% pulegone. A previous GC-MS study [28] of *M. satureioides* (identified as *M. diemenica*) that also examined samples from Didcot Queensland identified high levels of menthone (32%) and pulegone (25%), with neomenthyl acetate (18%) and menthyl acetate (6%) also found in substantial but lower quantities. Similarly, an earlier study using pure oil extract [27] showed that Dalby Queensland populations (only 30 km south of our JB6185—Jimbour West population) have high pulegone (40%) and l-menthone (20–30%), although our extraction demonstrated nearly equal quantities of menthone and pulegone (Table 7).

The remainder of samples we extracted from the New South Wales Central Coast, Central Tablelands, and (in part) North Coast botanical districts show a remarkably different chemotype. This second *M. satureioides* chemotype is distinct because it is consistently dominated by limonene (~17%) and 1,8-cineole (~19%), whereas menthone and pulegone were either not detected, or detected in low concentrations (<1%). Material from all commercial suppliers (2) that we sampled from exhibited this second chemotype.

### 3.3. Mentha atrolilacina Oil Profile

**Figure 13:**
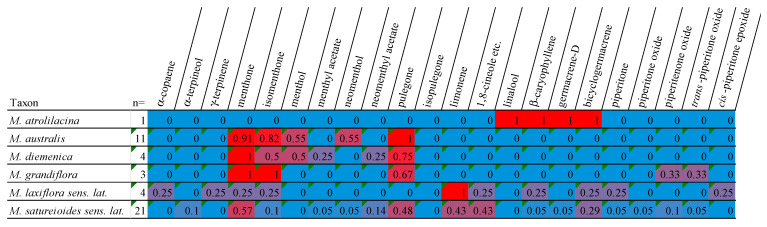
Heatmap identifying the variation of main volatile compounds detected for Australian *Mentha* as reported in Table 1, which demonstrates a summary of results from this study and the previous studies [22,28]. Numbers represent a percentage of the main volatile oils found within each species. For each species, this was calculated as the count of samples where a given oil is present, divided by the total count of samples (i.e., ‘n =’).

*Mentha atrolilacina* has the most distinctive profile of volatile oils compared to any other Australian *Mentha* species (Figure 13). Bicyclogermacrene is the principal component highest in quantity (24%) and found in lesser quantities across all other species of *Mentha* we examined. This species also has a nearly similar concentration of linalool (21%), a compound found highest in concentration in some non-Australian species such as *M. citrata* [40]. This chemical is not found in high quantities in any other Australian species from our result, and from previous studies [22,28]. One exception is that of ex situ material of *M. australis* from which linalool was identified as the most dominant compound [26].

### 3.4. Mentha laxiflora Oil Profile

*Mentha laxiflora* (like *M. satureioides*) also has variability in its main volatile compounds across the range we examined. The fresh material we examined from Tallaganda National Park in the Southern Tablelands of New South Wales (JB6231) has, as main components, piperitone and *cis*-piperitone epoxide, the latter also found in higher quantities for *M. grandiflora* [22]. Similar to menthone and pulegone, such compounds equally have a smell traditionally considered as ‘minty’. Hence, although no chemical compounds have previously been analysed, early reports about the ‘minty’ smell of *M. laxiflora* extracts at the 1861 Dublin Exhibition [2] correspond with our results. However, the population we examined in South Australia (JB6224) has a notably distinct oil profile with limonene (28%) as a principal component (with the next highest component being β-pinene, 12%). Our broader screening across the known distribution of *M. laxiflora* using herbarium specimens did indeed identify that limonene is a major component consistent across all populations; yet, this was accompanied by a combination of other compounds unique to each sample site. Hence, given the poor signal delivered in analyses of herbarium specimens, a reanalysis of fresh material would be necessary to confirm the exact quantities across this variation. Menthone was the only other component we detected that would produce a ‘minty’ smell corresponding with the early anecdotal evidence, but we only found it in the Goodradigbee River specimen (JB2618), proximally located to the sample from Tallaganda National Park in the Southern Tablelands of New South Wales.

### 3.5. Taxonomic Insights and Conclusion

We found variations in the dominant essential oils across Australian *Mentha* through sampling replicates for most species, and that not all of them have the main oil components corresponding to ‘mint-like’ smells as traditionally thought (e.g., menthone or pulegone). All replicates of *M. australis*, *M. diemenica,* and *M. grandiflora* yield such a ‘mint-like’ profile, whereas not all populations of *M. atrolilacina, M. laxiflora,* and *M. satureioides* do. *Mentha laxiflora* and *M. satureioides* have limonene in higher quantities for all replicate populations. However, other compounds in a high quantity vary between *M. laxiflora* populations, whereas they form two consistent chemotypes in *M. satureioides*.

Two distinct chemotypes within *M. satureioides* might merely be variations in the oil composition, since the quantities of oil may be expressed within different individuals of a species based on the genetic variation, ontogenetic processes, and environmental variation [34,41]. Despite some level of obfuscation, chemovariation can often align with phylogenetic signals such as interspecific boundaries [37]. Hence, such a discrete difference in the chemotypes of *M. satureioides* could indicate different taxa, especially since the high menthone and pulegone chemotype is found in similar woodland habitats as the high limonene chemotype, but, yet, is found in a broader, overlapping climatic envelope that also spans the well-recognised biogeographic barrier of the Great Dividing Range [42]. Considering the number of ambiguities currently associated with the ‘*M. satureioides* group’, any taxonomic conclusion is premature and a robust phylogenetic investigation is needed before the future identification of chemotaxonomic markers can be accomplished. Such an examination would require genomic data with a population-based sampling approach for all species, with special attention towards representing the morphological and chemical variation of the ‘*M. satureioides* group’ and *M. laxiflora*, and any instances of sympatry (e.g., *M. diemenica* and *M. atrolilacina* at Honans NFR) that could potentially reveal the levels of gene flow and reproductive isolation.

For the industry actively searching for Australian sources of ‘mint-like’ flavouring, our results so far support that *M. australis* is the best option. We confirm high quantities of menthone and pulegone are in *M. australis*, though the toxic nature of pulegone should be taken into account, and this is consistent across all wild populations and the wide number of plants we obtained from the horticultural trade. Populations currently identified as *M. diemenica* or *M. satureioides* might be useful as well; yet, we are hamstrung by the current lack of a robust taxonomy that would otherwise assist us with diagnosing phylogenetic patterns in chemovariation. Such a phylogenetic study would permit a more informed approach towards selecting plants for desired flavours (e.g., by pinpointing species highest in menthones), or low toxicity (e.g., by pinpointing species with lower levels of pulegones). A sound taxonomic framework is fundamental to equipping such downstream research endeavours, just as it is a necessity for the conservation of our biological heritage.

## 4. Materials and Methods

### 4.1. Plant Material

Morphology of plants was examined in the field and in cultivation. Specimens were consulted at the National Herbarium of NSW (NSW). Leaf material was collected from freshly collected, air-dried plants, or from herbarium specimens (Table 1). Herbaria acronyms are listed here according to Index Herbarium [43], and we identify botanical regions in New South Wales [44,45] according to the defined areas based on the National Interim Biogeographic Regionalisation for Australia system IBRA v7 [46]. Since similar studies have sampled from herbarium samples and shown the success of the technique [38,39,47], we sampled from herbarium samples at NSW that ranged from between 36 and 50 years old. *Mentha australis* and *M. satureioides* leaf oil were each extracted from fresh samples obtained through three different commercial suppliers, with the provenance of only one sample (JB6187) known to be from near Taree, New South Wales.

Robert Brown’s type material of *M. satureioides* (BM 000838674) was collected from Parramatta, New South Wales [48] and has mostly elliptic to ovate leaves that often have a slightly toothed margin—this is consistent with all NSW-held samples of the species collected from Queensland and the New South Wales Central Coast botanical district, as well as other specimens from the South Coast and North Coast botanical districts. However, specimens identified as *M. satureioides* that are found across this distribution and that resemble the type material can vary in the characters used to distinguish them in the *Mentha* key of the NSW and Victoria flora [15,16]. That is, there is a gradient in hair density of branches between individuals (i.e., from glabrous, or nearly so, to hairy), leaf lamina can range between linear and ovate, and the leaf margin can be entire to shallowly toothed on the same plant. Such morphological variation complicates the final couplet distinguishing the two taxa in the *Mentha* key in the NSW and Victoria flora [15,16].

There are also complications with diagnostic characters used in identification keys as revealed by our examination of the *M. diemenica* holotype (syn. *M. gracilis* R.Br.) made also by Robert Brown (BM 001041075). Though the NSW and Victoria Flora accounts describe *M. diemenica* as having ovate to angular-ovate leaves that correspond with Brown’s type material, their identification of plants with glabrous stems (c.f., hair) and often-toothed leaf margins is inconsistent with this, as well as with other material from New South Wales, Victoria, South Australia, and Tasmania.

For this study, we abandon the use of the stem indumentum as a diagnostic character and rely solely on matching leaf shape and marginal teeth on the leaf lamina with original material of *M. diemenica* (ovate, and often angular-ovate) (Figure 2g). For the purpose of this paper, we use *M. satureioides* sens. lat., referring to specimens that have linear leaves with an entire margin to ovate leaves with a shallowly toothed margin (e.g., Figure 2c–e).

A number of specimens have been identified as *M. laxiflora* on both sides of the South Australia–Victorian Border, the western-most extent of the species’ distribution [49]; yet, they do not closely resemble the type specimen made by Hooker (an unknown locality of Australia; K 000494937). For instance, our specimen JB6182 from this area (Figure 2h) in comparison to material resembling the type specimen (Figure 2i), has leaves less than 10 mm long (c.f., up to 30 mm long) and an entire to shallow-toothed margin (c.f. serrate). It furthermore has calyx lobes whose inner surface is glabrous, rather than having villous or spreading hairs as indicated by [15,16]. Hence, due to the distinctively different morphology, we identify this specimen as *M.* sp. aff. *laxiflora*.

### 4.2. Extraction of Volatile Components

Extraction of the volatiles was from the leaves. A small amount of leaf material (three or four leaves, between 0.05 g and 0.1 g fresh weight, or 0.02 g and 0.05 g dry weight) was placed in a two ml screw top vial, and then immersed in one ml pentane and sealed. The solution was kept at room temperature for at least two days and shaken at least once a day, and then the pentane solution was analysed by GC/MS as one sample of a batch run. As well as the extract, samples also included a solvent blank and a sample containing a solution of C10–C25 alkanes, to allow linear retention index (LRI) data to be calculated. From the strength of the TIC traces, the oil yields appeared to be poor.

### 4.3. Analysis of the Pentane Extract

The chief aim of this study was solely to acquire and compare relative rather than absolute amounts of volatile organic compounds and to identify the chief compounds in each *Mentha* specimen’s oil profile. The response from the Total Ion Current (TIC) trace is similar for all compounds found in this work because they, with very few exceptions, are all aliphatic/olefinic organic compounds. Only two aromatic compounds (thymol and carvacrol) were found in the whole of this work, and these were present at low levels. Aromatic compounds, because of the stability of the ions readily formed in the mass spectrometer ion source, tend to give a larger signal than those from aliphatic or olefinic compounds. Therefore, the small TIC signals for thymol and carvacrol indicate that they were present in very low levels. We found the mass spectrometer response to be similar among the compounds detected in this survey. We think it valid to show and produce the relative integrated area counts on all the TIC peaks. The presence of γ-terpinene in some samples and the lack of its oxidised product, *p*-cymene, indicates that the samples were not oxidised.

Analysis of the extract was carried out by combined gas chromatography/mass spectrometry (GC/MS) on a Shimadzu GCMS-2020 mass spectrometer, operating at 70 eV ionisation energy, in the e.i. mode. The GC column used was a BP-20 column (30 m × 0.25 mm × 0.25 μm), programmed from 35 °C to 220 °C at 3 °C/min with helium (1 mL/min) as carried gas, and injector temperature was 250 °C. Then, 1 mL of pentane solution was injected with a 5:1 split ratio. Mass spectra were recorded in the electron impact (EI) mode at 70 eV, scanning the 41–450 *m/z* range. Interface and source temperatures were 250 °C and 220 °C, respectively, with a 1 scan/sec cycle rime. Compounds were identified by their mass spectra, comparison with Adams and Wiley libraries, and linear retention indices (LRI). The hydrocarbon standard solution (C10–C25) was made from individual alkanes, purchased from Aldrich Chemical Co. USA and Tokyo Kasai Chemical Co. Tokyo. They were dissolved in pentane, ~10 mg/10 mL. As the number of volatiles in the herbarium samples was usually very small, the associated peaks, as opposed to the peaks from the impurities in the pentane solvent, were of comparable height. In these cases, the peaks in the TIC runs were examined individually to ensure that only compounds arising from the sample were reported on relative to n-alkanes [50,51] and by comparison of their mass spectra with either known compounds or published spectra [52] and Wiley libraries.

Every sample was analysed by GC/MS. For each species, these TIC traces were visually compared to find traces that lacked significant solvent impurity peaks. The chosen TIC trace was then manipulated to remove the response caused by the solvent impurity (even though the peak still remained on the TIC trace). The original TIC trace was then printed, but the peak areas reported from the result of removal of the impurity. The chart is characteristic of the TIC traces of that species or of the chemotype of that species. 

## Figures and Tables

**Table 1:** Specimen details of *Mentha* and corresponding GC/MS (gas chromatography/mass spectrometry) output used in this and previous studies. The main components column lists principal components in a trace, referring to the highest peaks from the trace. Samples analysed from freshly dried material (vs. samples removed from herbarium vouchers) are marked with an asterisk (*). Where there can be two chemotypes, [P/M] refers to the pulegone/menthol chemotype and [L/C] refers to the limonene/1,8-cineole chemotype. Australian states are abbreviated as NSW (New South Wales), Queensland (Qld), SA (South Australia), and Vic (Victoria).

Species	Oil Sample Number	Collector	Collector Number	Location (Number on Figure 3)	Locality Description	Main Components
* *M. atrolilacina*	JB6181	D. Duval	s.n.	1	Honans Native Forest Reserve, SA.	linalool, β-caryophyllene, germacrene-D, bicyclogermacrene
* *M. australis*	JB6172	J. Brophy	s.n.	provenance unknown	Cultivated, Tuckerbox, WA. Wild Source: Unknown.	menthone, isomenthone, neomenthol, pulegone, menthol
* *M. australis*	JB6174	J. Brophy	s.n.	provenance unknown	Cultivated, Indigigrow, Malabar, NSW. Wild Source: Unknown.	menthone, isomenthone, neomenthol, pulegone, menthol
* *M. australis*	JB6175	J. Brophy	s.n.	provenance unknown	Cultivated, Melbourne Bush Foods, Vic. Wild Source: Unknown.	menthone, isomenthone, neomenthol, pulegone, menthol
* *M. australis*	JB6177	J. Brophy	s.n.	provenance unknown	Cultivated, Greenpatch Organic Seeds and Plants, Taree, NSW. Wild Source: Unknown.	menthone, isomenthone, neomenthol, pulegone, menthol
* *M. australis*	JB6179	T.C. Wilson	s.n.	provenance unknown	Cultivated, Connells Point, NSW. Wild Source: Unknown.	menthone, isomenthone, neomenthol, pulegone, menthol
* *M. australis*	JB6180	T.C. Wilson	1147	2	Cultivated, Connells Point, NSW. Wild Source: Hall Reserve, Clifton Hill, Vic.	menthone, isomenthone, neomenthol, pulegone, menthol
*M. australis*	JB6213	K. Wilson	1783	5	Narran River on Goodooga-Lightning Ridge Road, NSW.	menthone, pulegone
*M. australis*	JB6219	J.T. Waterhouse UNSW	11050	7	Kennedy Developmental Road, Diamantina Crossing, western-most channel, Qld.	menthone, isomenthone, pulegone
*M. australis*	JB6220	P.L. Milthorpe	968	3	Menindee, NSW.	menthone, isomenthone, pulegone
*M. australis*	JB6221	J.L. Porter	29	4	Momba Swamp, 15 km S of Wanaaring, Nocoleche Nature Reserve, NSW.	menthone, isomenthone, pulegone
*M. australis*	JB6222	G.M. Cunningham & P.L. Milthorpe	3140	6	Lake Eyre, Alberga Creek near Alberga Station, SA.	pulegone
*M. diemenica*	JB6204	J. Strudwick	276	10	Northern end of Green Road, Upper Lurg. North East Study Area, Sector B, rr., Vic.	menthone, isomenthone, neomenthyl acetate, menthol [M/P]
*M. diemenica*	JB6205	V. Stajsic	1096	9	Tullich Swamp, at Tullich. At junction between Tullich Rd. & Corndale Rd. ca. 9 km from the junction between Tucllich R. & Glenelg Hwy (NE corner of swamp), Wannon, Vic.	menthone, pulegone very small amt. [M/P]
* *M. diemenica*	JB6209	D. Duval	s.n.	8	Honans Nature Native Forest Flora Reserve, SA.	menthone, pulegone, menthol [M/P]
*M. diemenica*	JB6212	D. Medd	160266	11	7 km from Orange on Cargo Road, NSW.	menthone, isomenthone, menthyl acetate, pulegone [M/P]
*M. grandiflora*	JB6202	P.I. Forster	PIF15084	12	Precipice National Park, Qld.	menthone, isomenthone, pulegone, “the oxides”
* *M. grandiflora*	JB3992 previously published [22]	P.I. Forster	PIF19721	12	Precipice National Park, Qld.	menthone, isomenthone, pulegone
*M. grandiflora*	JB6201	P.I. Forster	PIF24785	13	Bigge Range, NSW of Taroom, Turpentine Gorge, Palmgrove National Park, Qld.	menthone, isomenthone; very small peaks
*M. laxiflora*	JB6218	A.N. Rodd	846	16	Goodradigbee River, just above junction with Cave Creek (13 miles NE of Rules Point), Southern Tablelands, NSW.	limonene, α-copaene, β-caryophyllene
*M. laxiflora*	JB6224	J.R. Hosking	366	17	Warrabah National Park, Northern Tablelands, NSW.	limonene, 1,8-cineole, menthone, isomenthone, unknown mw 170
* M. laxiflora	JB6231	T.C. Wilson	1177	15	Tallaganda National Park, NSW.	*cis*-piperitone epoxide, piperitone, limonene
* *M. satureioides*	JB6173	J. Brophy	s.n.	provenance unknown	Cultivated, Tuckerbox, WA. Wild Source: Unknown.	limonene, 1,8-cineole, etc. [L/C]
* *M. satureioides*	JB6183	D. Duval	s.n.	18	Cultivated. Wild Source: Back Callington Road, Kanmantoo Kanmantoo, SA.	menthone, isomenthone, pulegone [M/P]
* *M. satureioides*	JB6184	D. Duval	s.n.	19	Cultivated. Wild Source: Tanunda Creek Road, Flaxman Valley Tanunda Creek, SA.	menthone, isomenthone, pulegone [M/P]
* *M. satureioides*	JB6185	P.I. Forster	PIF49091	30	Warra-Marnhull Road, in road reserve; Jimbour West, Darling Downs, Qld.	menthone, pulegone [M/P]
* *M. satureioides*	JB6186	P.I. Forster	PIF48899	29	Iredale Stud Farm, Monkey Water Holes Creek. Iredale, Lot/Plan: 2/RP32743, Moreton, Qld.	menthone, pulegone, piperitone, piperitenone oxide, *trans*-piperitone- oxide [M/P]
* *M. satureioides*	JB6187/JB6176	J. Brophy	s.n.	26	Cultivated, Greenpatch Organic Seeds and Plants, Taree, NSW. Wild Source: Taree, NSW.	limonene, 1,8-cineole, [L/C]
* *M. satureioides*	JB6189	P.I. Forster	PIF49351	31	Boyne River Water Reserve, off Kingaroy—Burrandowan Road; Lot/Plan: 63/BO98, Chahpingah, Burnett, Qld.	menthone, pulegone [M/P]
* *M. satureioides*	JB6190	P.I. Forster	PIF49107	33	‘Nora Creina’, 1.5 km NE of Didcot; Lot/Plan: 13/CK79, Didcot, Wide Bay, Qld.	menthone, pulegone [M/P]
* *M. satureioides*	JB6191	P.I. Forster	PIF49359	28	McEwan State Forest, 5.5 km N of Pittsworth, Darling Downs, Qld.	menthone, pulegone [M/P]
* *M. satureioides*	JB6203	P.I. Forster	PIF20147	34	Round About Scrub, Dry Creek, State Forest 316, Kroombit, Qld.	menthone, pulegone, spathulenol [M/P]
* *M. satureioides*	JB6206	C.P. Gibson	s.n.	22	Cumberland Plain Woodland at Lansdowne, Central Coast, NSW.	limonene, 1,8-cineole, β-caryophyllene, bicyclogermacrene [L/C]
*M. satureioides*	Jb6207	T.C. Wilson	456	36	Inverell, NSW.	menthone, neomenthyl acetate, pulegone [M/P]
*M. satureioides*	JB6208	J.J. Bruhl	2067	27	Armidale, University of New England campus, North Hill, 15 m north of Ring Rd, under power lines.	menthone, piperitone oxide, piperitenone oxide [M/P+]
*M. satureioides*	JB6210	O.R. Semple	17909	25	Daydawn, c.5 km NNE of Kerrs Creek, Central Western Slopes, NSW.	limonene, 1,8-cineole, bicyclogermacrene [L/C]
*M. satureioides*	JB6211	B.S. Wannan	3128	35	South of Ravenshoe, North Kennedy, Qld.	menthone, neomenthyl acetate, menthyl acetate, bicyclogermacrene [M/P]
* *M. satureioides*	JB6216/7094	T.C. Wilson	1050	21	Cultivated. Wild Source: Collection TW1019. Duck River, Wategora Reserve, NSW.	limonene, 1,8-cineole [L/C], bicyclogermacrene
*M. satureioides*	JB6217	J. Thompson	s.n.	24	Euroka Clearing, 2.5 miles south of Glenbrook, Central Tablelands, NSW.	limonene, 1,8-cineole, germacrene-D, bicyclogermacrene [L/C]
* *M. satureioides*	JB6228	T.C. Wilson	1144	20	Cumberland Woodland nearby staff carpark, Australian Botanic Garden, Mount Annan, NSW.	limonene, 1,8-cineole, α-terpineol, bicyclogermacrene [L/C]
* *M. satureioides*	JB6229	T.C. Wilson	1145	23	Annan Creek, Australian Botanic Garden, Mount Annan, NSW.	limonene, 1,8-cineole, [L/C]
* *M. satureioides*	JB6230	T.C. Wilson	1146	provenance unknown	Cultivated. A2021-9170/2, Bed 213, Australian Botanic Garden, Mount Annan, NSW. Wild Source: Unknown-source Wildtech Nursery Pty Ltd.	limonene, 1,8-cineole [L/C]
* *M. satureioides*	previously published [23]	P.I. Forster	PIF15084	32	Didcot, Qld.	menthone, neomenthyl acetate, neomenthol, pulegone [M/P]
* *M.* sp. aff *laxiflora*	JB6182	D. Duval	s.n.	14	Track to Dry Creek campground, Dry Creek Road. Limestone overhang adjacent road to shacks, approximately 4 km north of Donovans, SA.	limonene, γ-terpinene, germacrene-D, bicyclogermacrene

*Sine nomine* (s.n.) is written where a collecting number does not exist.

**Table 2:** Volatile compounds identified in the leaf extract of *Mentha atrolilicana* (sample JB6181, Table 1). An asterisk (*) indicates where an isomer was not identified. Compounds, in this and following tables, are listed in order of elution from a polar GLC column. **Σ** indicates the sum of the individual percentages, in **bold type** to differentiate from the individual values.

#	Compound	LRI Range	LRI Found	Amount Present (%)
1	octyl acetate	1365–1402	1380	0.4
2	hex-3-en-1-ol *	1344–1399	1385	0.3
3	octan-3-ol	1372–1408	1398	0.2
4	bicycloelemene	1471–1495	1441	0.3
5	1-octen-3-ol	1411–1465	1456	0.5
6	γ-elemene	1471–1495	1463	4.9
7	α-copaene	1462–1495	1467	1
8	β-bourbonene	1496–1546	1493	0.3
9	benzaldehyde	1481–1555	1510	0.5
10	β-copaene	1550–1603	1547	0.1
11	linalool	1507–1564	1552	21.2
12	β-caryophyllene	1569–1632	1568	14.1
13	β-elemene	1565–1608	1572	0.1
14	aromadendrene	1583–1668	1616	3.7
15	α-humulene	1637–1689	1640	0.3
16	germacrene-D	1676–1726	1680	13.8
17	bicyclogermacrene	1192–1757	1704	23.7
18	δ-cadinene	1722–1774	1737	1.1
19	2-phenylethyl alcohol	1859–1944	1907	0.3
20	germacrene-D-4-ol	2000–2070	2033	1.9
**Σ**				**88.7**

**Table 3:** Compounds identified in the extract of *Mentha australis* from a commercial sample with unknown provenance (sample JB6174, Table 1). An asterisk (*) indicates where an isomer was not identified. **Σ** indicates the sum of the individual percentages, in bold type to differentiate from the individual values.

#	Compound	LRI Range	LRI Found	Amount Present (%)
1	α-pinene	1008–1039	1010	1.3
2	hexanal	1056–1106	1078	0.4
3	β-pinene	1085–1130	1088	1
4	sabinene	1098–1140	1092	0.6
5	limonene	1148–1219	1136	0.2
6	1,8-cineole	1186–1231	1146	0.1
7	hex-2-enal *	1196–1249	1207	1.5
8	hex-3-enyl acetate *	1276–1335	1275	0.4
9	3-hexene-1-ol *	1344–1398	1371	2.2
10	menthone	1443–1479	1442	31.5
11	isomenthone	1464–1479	1470	11
12	benzaldehyde	1481–1555	1513	0.5
13	*cis*-isopulegone	1587	1555	0.7
14	*trans*-isopulegone	1592–1598	1568	0.5
15	neomenthol	1551–1604	1594	2.3
16	pulegone	1626–1663	1626	31
17	menthol	1599–1651	1638	3.1
18	germacrene-D	1675–1726	1678	0.6
19	α-terpineol	1654–1724	1695	0.6
20	bicyclogermacrene	1692–1757	1702	1.9
21	δ-cadinene	1722–1774	1735	0.2
22	methyl salicylate	1727–1785	1767	0.5
23	benzyl alcohol	1821–1905	1881	0.3
24	piperitenone	1840–1949	1887	0.3
25	germacrene-D-4-ol	2000–2070	2032	5.1
	**Σ**			**97.8**

**Table 4:** Compounds identified in the extract of *Mentha diemenica* from Honans Native Forest Reserve, South Australia (JB6209); see Table 1 for specimen details. **Σ** indicates the sum of the individual percentages, in bold type to differentiate from the individual values.

#	Compound	LRI Range	LRI Found	Amount Present (%)
1	α-pinene	1008–1039	1020	1
2	β-pinene	1056–1106	1105	0.9
3	sabinene	1098–1140	1092	0.6
4	myrcene	1140–1175	1160	1.1
5	limonene	1140–1175	1180	7.2
6	1,8-cineole	1186–1231	1188	0.1
7	E-β-ocimene	1232–1267	1247	0.8
8	menthone	1443–1479	1439	32.5
9	isomenthone	1464–1479	1467	4.1
10	neomenthyl acetate	1569	1517	5.2
11	*cis*-isopulegone	1587	1551	2.5
12	neomenthol	1551–1604	1592	0.6
13	pulegone	1626–1663	1621	29.8
14	menthol	1599–1651	1635	9.7
15	germacrene-D	1675–1726	1678	0.7
16	α-terpineol	1654–1724	1691	0.3
17	piperitone	1689–1748	1701	0.1
18	bicyclogermacrene	1692–1757	1702	0.5
19	carvone	1699–1751	1704	0.4
20	δ-cadinene	1722–1774	1735	0.1
21	piperitenone	1840–1949	1894	0.5
22	germacrene-D-4-ol	2000–2070	2037	0.3
23	spathulenol	2074–2150	2138	0.9
	**Σ**			**99**

**Table 5:** Compounds identified in the extract of *Mentha laxiflora* from Tallaganda National Park, New South Wales (sample JB6231). **Σ** indicates the sum of the individual percentages, in bold type to differentiate from the individual values.

#	Compound	LRI Range	LRI Found	Amount Present (%)
1	α-pinene	1008–1039	1010	0.8
2	β-pinene	1085–1130	1089	1.1
3	sabinene	1098–1092	1160	1
4	myrcene	1140–1175	1161	2.2
5	1,4-cymene	1169–1212	1700	0.6
6	limonene	1148–1219	1183	9
7	1,8-cineole	1186–1231	1186	3.5
8	C_10_H_16_ (unknown)		1224	1.5
9	γ-terpinene	1221–1266	1234	2
10	p-cymene	1246–1291	1254	4.1
11	aromadendrene	1583–1668	1606	1.5
12	germacrene-D	1657–1726	1680	1.9
13	*cis*-piperitone epoxide	1707	1690	30.4
14	piperitone	1689–1748	1699	24.9
15	bicyclogermacrene	1692–1757	1705	2.1
16	*trans*-piperitone epoxide	1727	1707	7.4
17	δ-cadinene	1722–1774	1736	0.3
18	isopiperitenone (tent)	1833–1865	1816	0.2
19	thymol	2100–2205	2193	5
20	carvacrol	2140–2246	2218	0.4
	**Σ**			**99.5**

**Table 6:** Compounds identified in the extract of *Mentha* sp. aff. *laxiflora* from Dry Creek Road, north of Donovans SA (sample JB6182). An asterisk (*) indicates the isomer is not identified. **Σ** indicates the sum of the individual percentages, in bold type to differentiate from the individual values.

#	Compound	LRI Range	LRI Found	Amount Present (%)
1	α-pinene	1008–1039	1010	5.8
2	hexanal	1056–1106	1078	0.5
3	β-pinene	1085–1130	1088	11.3
4	sabinene	1098–1140	1092	6.3
5	myrcene	1140–1175	1117	0.1
6	limonene	1148–1219	1136	27.4
7	β-phellandrene	1188–1233	1144	1.4
8	1,8-cineole	1186–1231	1146	0.1
9	2-hexenal	1196–1249	1207	4.4
10	γ-terpinene	1221–1266	1195	7.2
11	hex-2-en-1-ol acetate *	1315–1348	1325	0.4
12	hex-3-en-1-ol *	1344–1399	1371	2.2
13	hex-2-en-1-ol *	1377–1419	1385	0.6
14	bicycloelemene	1471–1495	1450	1.2
15	α-copaene	1462–1495	1456	2.6
16	β-copaene	1550–1603	1499	0.9
17	linalool	1507–1564	1550	1
18	β-elemene	1565–1608	1560	0.5
19	aromadendrene	1583–1668	1604	0.3
20	germacrene-D	1375–1726	1678	4.5
21	borneol	1653–1728	1689	0.8
22	bicyclogermacrene	1692–1757	1702	7.1
23	δ-cadinene	1722–1774	1735	1.2
24	germacrene-D-4-ol	2000–2070	2032	0.9
25	eugenol	2100–2198	2062	0.3
	**Σ**			**89**

**Table 7:** Compounds identified in the extract of *Mentha satureioides* from Jimbour West, Queensland (JB6185) and Kanmantoo, South Australia (JB6183). An asterisk (*) indicates the isomer is not identified. **Σ** indicates the sum of the individual percentages, in bold type to differentiate from the individual values.

#	Compound	LRI Range	LRI Found	Amount Present (%)	Amount Present (%)
				Sample JB6185	Sample JB6183
1	α-pinene	1006–1039	1010	1.1	
2	β-pinene	1085–1130	1040	0.7	1.5
3	sabinene	1098–1140	1058	0.5	0.6
4	myrcene	1140–1175	1160	1.3	2.9
5	limonene	1178–1219	1180	1.2	2.5
6	1,8-cineole	1186–1231	1188	0.3	0.2
7	E-β-ocimene	1232–1267	1247		2.7
8	octanal	1267–1312	1282	0.3	
9	isoamyl isovalerate	1284–1304	1295		0.4
10	unknown, mw 152, see below for MS		1361		1.2
11	3-hexen-1-ol *	1344–1399	1371		0.3
12	menthone	1443–1479	1439	22.4	36.8
13	isomenthone	1464–1503	1467	8.7	6.7
14	decanal	1471–1511	1491	0.9	
15	neomenthyl acetate	1602	1517	7.2	
16	menthyl acetate	1535–1585	1551	4.7	
17	*cis*-isopulegone	1587	1551	3.5	0.8
18	linalool	1489–1543	1553		0.3
19	*trans*-isopulegone	1592–1598	1565		1.4
20	neomenthol	1551–1604	1592	3.1	1.5
21	pulegone	1626–1663	1621	22.5	12.1
22	menthol	1599–1651	1653	3.1	0.7
23	δ-terpineol	1655–1687	1678	0.2	
24	germacrene-D	1676–1726	1708		1
25	α-terpineol	1659–1724	1691	1.6	0.9
26	piperitone	1689–1748	1701	5.3	2.8
27	bicyclogermacrene	1692–1757	1702	0.2	2.2
28	isopiperitenone	1833–1865	1816	0.3	0.5
29	piperitenone	1840–1949	1894	2.4	0.7
30	piperitenone oxide	1940–1984	1938	0.2	0.6
31	germacrene-D-4-ol	2000–2070	2037		0.6
	**Σ**			**91.7**	**81.3**

**Mass Spectrum of Compound 10.** LRI 1361 for sample JB6183: 152 (M+, 35%), 137 (100%), 119 (17%), 110 (13%), 109 (15%), 95 (19%), 93 (21%), 91 (11%), 81(21%), 67 (30%), 55 (12%), and 41 (18%).

**Table 8:** Compounds identified in the extract column for *Mentha satureioides* from Wategora Reserve, NSW (sample JB6216, Figure 11). **Σ** indicates the sum of the individual percentages, in bold type to differentiate from the individual values.

#	Compound	LRI Range	LRI Found	Amount Present (%)
1	α-pinene	1008–1039	1010	1.3
2	β-pinene	1085–1130	1089	1
3	sabinene	1098–1140	1161	9.4
4	myrcene	1140–1175	1161	1
5	dehydro-1,8-cineole	1167–1197	1177	0.2
6	limonene	1178–1219	1182	17.3
7	1,8-cineole	1186–1231	1188	19.2
8	isoamyl alcohol	1179–1236	1211	1.6
9	γ-terpinene	1221–1266	1233	1.2
10	E-β-ocimene	1232–1267	1248	0.6
11	*p*-cymene	1246–1291	1256	0.3
12	terpinolene	1261–1291	1256	0.3
13	bicycloelemene	1471–1495	1460	0.8
14	α-copaene	1462–1495	1467	0.6
15	benzaldehyde	1481–1555	1511	0.2
16	neomenthyl acetate	1569	1517	0.8
17	menthyl acetate	1535–1585	1551	2.9
18	linalool	1507–1564	1554	0.4
19	β-caryophyllene	1568–1632	1569	1.7
20	terpinen-4-ol	1564–1630	1593	0.2
21	δ-terpineol	1655–1687	1667	2.2
22	germacrene-D	1692–1757	1706	0.3
23	α-terpineol	1654–1724	1693	8.1
24	piperitone	1689–1748	1701	2.2
25	bicyclogermacrene	1692–1757	1706	9.1
26	δ-cadinene	1722–1774	1738	0.2
27	unknown, mw 170, see below for MS		1855	6.2
	**Σ**			**89.1**

**Mass spectrum of unknown Compound 27.** LRI 1855: *m*/*z* 170 (M+. 5%), 155 (2%), 126 (45%), 111 (12%), 108 (100%), 93 (35%0, 83 (17%), 71 (54%), 69 (16%), and 43 (92%).

**Table 9:** Compounds identified in the essential oil of *Mentha grandiflora* collected from Precipice National Park, Queensland, as per results from the study by Brophy et al. [22]. **Σ** indicates the sum of the individual percentages, in bold type to differentiate from the individual values.

#	Compound	LRI Range	LRI Found	Amount Present (%)
1	α-pinene	1008–1039	1020	0.5
2	β-pinene	1056–1106	1105	0.6
3	sabinene	1098–1140	1120	0.4
4	an amyl acetate	1102–1140	1125	0.2
5	α-phellandrene	1148–1186	1150	0.1
6	myrcene	1140–1175	1156	0.5
7	limonene	1178–1219	1205	3.5
8	1,8-cineole	1186–1231	1208	0.1
9	E-β-ocimene	1232–1267	1254	0.1
10	terpinolene	1261–1291	1283	0.1
11	1-octen-3-yl acetate	1365–1402	1382	0.7
12	3-octanol	1372–1408	1395	0.1
13	menthone	1443–1479	1459	9.7
14	isomenthone	1464–1479	1486	0.1
15	β-caryophyllene	1569–1932	1594	0.4
16	terpinin-4-ol	1564–1630	1600	0.2
17	pulegone	1626–1663	1644	19.1
18	α-terpineol	1654–1724	1700	0.9
19	*cis*-piperitone oxide	1707	1707	0.3
20	*trans*-piperitone oxide	1727	1727	21.4
21	bicyclogermacrene	1692–1757	1732	2.1
22	iso-piperitenone	1833–1865	1836	0.8
23	piperitenone	1840–1949	1918	1.7
24	piperitenone oxide	1963	1963	36.2
	**Σ**			**98**

## Data Availability

The raw data (Shimadzu data system files) supporting the conclusions of this article will be made available by the authors upon request.
